# Comprehensive Multiplex One-Step Real-Time TaqMan qRT-PCR Assays for Detection and Quantification of Hemorrhagic Fever Viruses

**DOI:** 10.1371/journal.pone.0095635

**Published:** 2014-04-21

**Authors:** Zheng Pang, Aqian Li, Jiandong Li, Jing Qu, Chengcheng He, Shuo Zhang, Chuan Li, Quanfu Zhang, Mifang Liang, Dexin Li

**Affiliations:** Key Laboratory of Medical Virology, NHFPC, National Institute for Viral Disease Control and Prevention, China CDC, Beijing, China; Technical University of Braunschweig, Germany

## Abstract

**Background:**

Viral hemorrhagic fevers (VHFs) are a group of animal and human illnesses that are mostly caused by several distinct families of viruses including *bunyaviruses*, *flaviviruses*, *filoviruses* and *arenaviruses*. Although specific signs and symptoms vary by the type of VHF, initial signs and symptoms are very similar. Therefore rapid immunologic and molecular tools for differential diagnosis of hemorrhagic fever viruses (HFVs) are important for effective case management and control of the spread of VHFs. Real-time quantitative reverse transcriptase-polymerase chain reaction (qRT-PCR) assay is one of the reliable and desirable methods for specific detection and quantification of virus load. Multiplex PCR assay has the potential to produce considerable savings in time and resources in the laboratory detection.

**Results:**

Primers/probe sets were designed based on appropriate specific genes for each of 28 HFVs which nearly covered all the HFVs, and identified with good specificity and sensitivity using monoplex assays. Seven groups of multiplex one-step real-time qRT-PCR assays in a universal experimental system were then developed by combining all primers/probe sets into 4-plex reactions and evaluated with serial dilutions of synthesized viral RNAs. For all the multiplex assays, no cross-reactivity with other HFVs was observed, and the limits of detection were mainly between 45 and 150 copies/PCR. The reproducibility was satisfactory, since the coefficient of variation of Ct values were all less than 5% in each dilution of synthesized viral RNAs for both intra-assays and inter-assays. Evaluation of the method with available clinical serum samples collected from HFRS patients, SFTS patients and Dengue fever patients showed high sensitivity and specificity of the related multiplex assays on the clinical specimens.

**Conclusions:**

Overall, the comprehensive multiplex one-step real-time qRT-PCR assays were established in this study, and proved to be specific, sensitive, stable and easy to serve as a useful tool for rapid detection of HFVs.

## Introduction

Viral hemorrhagic fevers (VHFs) generally describe a severe, variety of viral often fatal diseases characterized by fever and bleeding in humans [1], [2], which are caused by several distinct families of enveloped, single-stranded RNA viruses including *Bunyaviridae*, *Flaviviridae*, *Filoviridae* and *Arenaviridae*, and a novel rhabdovirus associated with acute hemorrhagic fever found in Central Africa in 2012 [3]–[9]. After transmission from their reservoir hosts or vectors to humans, or even spread from person to person, many of hemorrhagic fevers viruses (HFVs) cause severe, life-threatening diseases [10], [11]. The clinical symptoms in the early phase of VHFs are very similar irrespective of the causative viruses and resemble a flu-like illness or common enteritis, often including marked fever, fatigue, dizziness, muscle aches, loss of strength, and exhaustion [12], . Therefore, it is too difficult to distinguish the various etiologic agents based on clinical signs and symptoms, which makes the accurate and timely laboratory detection of viruses important in early diagnosis of VHFs.

In view of its identifying the selected target gene of RNA viruses rapidly and specifically, probe-based real-time quantitative reverse transcription-polymerase chain reaction (qRT-PCR) assay is widely used for virus detection [14], [15]. Quite a lot of such methods for detection of hemorrhagic fever viruses have been published, which provides useful references for people working on VHFs [16]–[20]. However, most of these qRT-PCR assays may cover limited virus strains or apply under different cycling conditions. Therefore, a panel of reliable comprehensive one-step real-time qRT-PCR assays covering all important pathogens, suitable for multiplex screening or specific quantitative identification with fast turn-around time and identical cycling parameters is still urgently needed, so that the unknown samples can be tested simultaneously and effectively.

Here, we established a series of one-step real-time qRT-PCR assays for multiplex detection of 28 viruses, which covered nearly all the important viral pathogens that cause VHFs, including Hantaan virus (HTNV), Seoul virus (SEOV), Puumala virus (PUUV), Dobrava virus (DOBV), Tula virus (TULV), Black creek canal virus (BCCV), Andes virus (ANDV), Sin nombre virus (SINV), Crimean-congo hemorrhagic fever virus (CCHFV), Rift valley fever virus (RVFV), Severe fever with thrombocytopenia syndrome virus (SFTSV), Heartland virus (HLV), Omsk hemorrhagic fever virus (OHFV), Kyasanur forest disease virus (KFDV), Dengue virus (DENV), Yellow fever virus (YFV), Marburg virus (MARV), Ebola Zaire virus (ZEBOV), Ebola Sudan virus (SEBOV), Ebola Cote d’Ivoire virus (CEBOV), Junin virus (JUNV), Machupo virus (MACV), Guanarito virus (GTOV), Sabia virus (SABV), Chapare virus (CHAV), Lassa virus (LASV), Lujo virus (LUJV) and Bas-Congo virus (BASV). All assays were optimized at a universal thermal cycling condition, and evaluated under monoplex or multiplex condition for detection and absolute quantification of viral RNAs, which were proved to be reliable molecular tools of early diagnosis and consequently addressing the threat of viral hemorrhagic fevers.

## Results

### Selection of Primers and Probes

Genomic sequences of all representative strains of each viral species were downloaded from the GenBank database (Supplementary Table S1). Alignments were performed with Clustal W using complete sequences of listed HFVs. After visual inspection of the sequence alignments, targeted genomic regions with high conservation were chosen, which located at encoding gene of nucleocapsid protein (NP) of viruses from *Bunyaviridae*, *Filoviridae* and BASV, non-structural protein 5 (NS5) of viruses from *Flaviviridae*, and glycoprotein (GP) of viruses from *Arenaviridae*. In total, 27 primer-probe pairs were designed and appraised, and all primers/probes were grouped into seven groups based on the related diseases or virus families. Of them, ANDV and SINV were designed to share the same primers/probe set in Group B (Table 1). BLAST analysis was also performed among chose viral target sequences in NCBI database to confirm the specificities of the primer-probe sets.

**Table 1:** Primers, probes, and amplicon sizes of the one-step real-time qRT-PCR assays.

Group	Viruses	GenBankAccession No.	ForwardPrimers	ReversePrimers	Probes	Ampliconsize (bp)
**A**	**_HTNV_**	_NC_005218_	_F(771–793): GCTTCTTCCAGATACAGCAGCAG_	_R(862–884): GCCTTTGACTCCTTTGTCTCCAT_	_P(811–839): FAM-CCTGCAACAAACAGGGAYTACTTACGGCA-BHQ1_	_114_
	**SEOV**	NC_005236	F(217–237): GATGAACTGAAGCGCCAACTT	R(272–291): CCCTGTAGGATCCCGGTCTT	P(239–263): HEX-CCGACAGGATTGCAGCAGGGAAGAA-BHQ1	76
	**PUUV**	NC_005224	F(181–201): AGGCAACAAACAGTGTCAGCA	R(334–359): GCATTTACATCAAGGACATTTCCATA	P(278–304): Texas Red-CTGACCCGACTGGGATTGAACCTGATG-BHQ2	179
	**DOBV**	NC_005233	F(755–772): TGACCTCCCRTGCAARCT	R(801–818): GGTGGATGGGCCTTTGGT	P(777–803): Cy5-TCTGAGCCATCWCCAACRTCTTTGACC-BHQ2	65
**B**	**TULV**	NC_005227	F(181–199): AGACGGGCAGCTGTGTCAG	R(286–303): ATCCGGCTCAAGCCCAGT	P(215–239): Texas Red -GGCAGACTTCAAGAGGCAGCTTGC-BHQ2	126
	**BCCV**	L39949	F(818–842): CGACAAATGGTGCTTACTTTATGAA	R(884–909): TGATTCAGCAGTGTCAATTAGGTCTA	P(847–875): Cy5-CAGACACAGGTTGAAGAGTCAAAGGTGCA-BHQ2	92
	**ANDV/** **SINV**	NC_003466	F(81–102): ACACGAACAACAGCTCGTGACT	R(278–299): GGTTCAATCCCTGTTGGATCAA	P(197–224): FAM-CTRCATTGGAGACCAAACTCGGRGAACT-BHQ1	219
**C**	**CCHFV**	NC_005302	F(726–747): GCCGTTCAGGAATAGCACTTGT	R(869–889): TGTTATCATGCTGTCGGCRCT	P(750–777): HEX-CAACAGGCCTTGCYAAGCTYGCAGAGAC-BHQ1	164
	**RVFV**	NC_014395	F(1254–1275): CATGGTWGTCCCAGTGACAGGA	R(1330–1355): GATGAGTTGACTCTATCACGAGTTGC	P(1276–1303): Cy5-AGCCACTCACTCAAGACGACCARAGCCT-BHQ2	102
	**SFTSV**	HM745932	F(1104–1125): GGGTCCCTGAAGGAGTTGTAAA	R(1155–1178): TGCCTTCACCAAGACTATCAATGT	P(1127–1146): Texas Red -TTCTGTCTTGCTGGCTCCGCGC-BHQ2	75
	**HLV**	JX005842	F(1457–1484): GCATTCTCCTTCAGCTCATAGACTCTAG	R(1525–1549): GAACAAGATAGTGAAAGCATGTGGC	P(1495–1525): FAM-CATCCTCTCAGCGCCTTTCTTAGACATCTTG-BHQ1	93
**D**	**OHFV**	NC_005062	F(9557–9579): TCGAGGCTACAGACTCACACAAC	R(9614–9633): AAGGCGTTCTTCTCCGTGGT	P(9588–9611): FAM-CGCAGCCACCTCTCCACTCGTAGC-BHQ1	77
	**KFDV**	NC_004355	F(9416–9434): GAGGCTGCGTCATGGACAT	R(9487–9508): CCTTGATGTTCGTGAGGGTGTT	P(9451–9473): HEX-CAACGTGGTTCAGGCCAGGTGGT-BHQ1	93
	**DENV**	NC_001474	F(8977–9002): GGAAGTAGAGCAATATGGTACATGTG	R(9157–9179): CCGGCTGTGTCATCAGCATAYAT	P(9082–9109): Texas Red -TGTGCAGTCCTTCTCCTTCCACTCCACT-BHQ2	203
	**YFV**	NC_002031	F(9858–9878): GAGGAAGGGTGTCTCCAGGAA	R(9911–9932): ACATGTTGGCATAGGCTTTGCT	P(9883–9910): CY5-CTGGATGATCAAGGAAACAGCTTGCCTC-BHQ2	75
**E**	**MARV**	NC_001608	F(1179–1203): AACAATTCCACCTTCAGAAAACTGA	R(1310–1331): GTGACACCCGATTCTGTGATTG	P(1209–1236): Cy5-CACACAGTCAGACAYTNGCCGTCCTCAG-BHQ2	153
	**ZEBOV**	NC_002549	F(501–521): CGCCGAGTCTCACTGAATCTG	R(609–633): AGTTGGCAAATTTCTTCAAGATTGT	P(578–608): FAM-CGGCAAAGAGTCATCCCAGTGTATCAAGTAA-BHQ1	133
	**SEBOV**	NC_006432	F(1832–1855): GTTGACCCGTATGATGATGAGAGT	R(1915–1934): CATCGTCGTCGTCCAAATTG	P(1870–1894): HEX-CTACGAGGATTCGGCTGAAGGCACC-BHQ1	103
	**CEBOV**	NC_014372	F(2030–2051): CGAAACCCGACTAATATGCCAA	R(2095–2117): TGTTATCCCTGGCGTATTCTTGA	P(2055–2085):Texas Red -AAGACTCCACACAAAACAATGACAATCCTGC-BHQ2	88
**F**	**JUNV**	NC_005081	F(1135–1158): TGATGAGTGTCCCYTACTGCAATT	R(1251–1276):AATATCCAGTCATTACGGAAGTCAGA	P(1192–1220): FAM-CAGGACAACACTCATTRCCAAGGTGCTGG-BHQ1	142
	**MACV**	NC_005078	F(1388–1411): GTTGAYATTTGTTTCTGGAGCACA	R(1478–1497): TGAGGCAAAGGACAGGCTTC	P(1456–1479): HEX-CACCCATCGACACCTCAAAGGCGA-BHQ1	110
	**GTOV**	NC_005077	F(847–867): TGACATGCCAGGTGGTTACTG	R(919–941): TCAAATTACACTTGGCRACAGCT	P(865–893): Texas Red -CAGCAACCAACATCCACCTYTCAAGACAG-BHQ2	95
	**SABV**	NC_006317	F(882–905): TTGAAAGATGGATGCTAGTGACGT	R(961–983): TCAACATGTCACAGAATTCCGAA	P(928–959): Cy5-CACAGCACTAGCAAAATGTAACCTTGACCACG-BHQ2	102
**G**	**CHAV**	NC_010562	F(1109–1133): GACACTCCCTACTGCAACTACACAA	R(1176–1196): TAACCATCCAACAGCGTGGAA	P(1144–1171): HEX-TGTCAACCACACCATCACAGGAGAGCAT-BHQ1	88
	**LASV**	NC_004296	F(2380–2402): ATGGCTTGTTTGTTGAAGTCRAA	R(2488–2509): TGACCAGGTGGATGCTAATTGA	P(2412–2442): Texas Red -CATGTCACAAAATTCTTCATCGTGCTTCTCA-BHQ2	130
	**LUJV**	NC_012776	F(571–591): GGCCCATGATGACAAGAACTG	R(637–661): CCTCACTTTGTAGTGGGTTTCTGAA	P(599–628): Cy5-CTACACCCATTGAACTACCTGAGGCTCCTG-BHQ2	91
	**BASV**	JX297815	F(763–785): GGACTGGGATTGGTCACTAGGTC	R(842–865): TGGATCTGTCTGAATGAAGGACTG	P(794–819): FAM-CTGCATCGGCCTGTTCCAACCTGTAC-BHQ1	103

### Preparation of Viral RNA Standards

To evaluate the designed primers/probe and optimize the real-time PCR assay, viral RNA standards were needed. Firstly double-stranded DNA fragments of the complete open reading fragments (ORFs) of NP coding gene of the viruses from *Bunyaviridae* and *Filoviridae*, partial ORF of NP coding gene of BASV, complete ORFs of GP coding gene of the viruses from *Arenaviridae,* and partial ORFs of NS5 coding gene of the viruses from *Flaviviridae* were obtained through chemical synthesis or RT-PCR amplification from viral isolates (HTNV, SEOV, SFTSV, DENV and YFV). After the introduction of T7 promoter sequence to the 5′ or 3′ terminus of these DNA fragments using PCR amplification, the PCR products were used as DNA templates for *in vitro* transcription of positive- or negative-sense viral RNA standards corresponding to related viruses. The resulted 28 RNA transcripts were purified and measured by NanoDrop Spectrophotometer. The 260 nm/280 nm ratios were all between 2.0 and 2.1, indicating that the RNA products were highly pure. The concentration of RNA transcripts were quantified with ranging from 200 to 1185 ng/µL, and the copy numbers were calculated respectively according to the concentration and size of each single-stranded RNA fragment (Supplementary Table S2). Then, a serial dilution (10^1^ to 10^8^ copies/µL) of purified viral RNA transcripts was used to create RNA standard templates for development of one-step real-time qRT-PCR.

### Development of One-step Real-time qRT-PCR Assays

The goal was to obtain one universal system able to detect effectively all 28 common hemorrhagic fever viruses listed above. Thus we performed numerous assays to optimize the concentrations of primers/probe and experimental condition, which consequently resulted a developed one-step real-time RT-PCR assay.

The optimized reaction system of 25 µL total volume consisted of 12.5 µL of 2× RT-PCR buffer, 400 nM of each primer, 120 nM of each probe, 1 µL of Enzyme Mix and 5 µL of viral RNA transcripts or RNA extracts. The optimal reaction conditions of a one-step assay were used as follows: 50°C for 30 min, 95°C for 10 min, then 40 cycles of 15 s at 95°C and 45 s at 60°C. The CT value for a positive sample was set at 35 cycles according to a liner range of a typical standard curve for each virus detection assay, which was also a strict standard for positive determination.

Using the developed reaction system, we tested each primers-probe set in the monoplex assays, and then combined them into 4-plex reactions for multiplex one-step real-time qRT-PCR assays according to Table 1.

### Specificities and Qualitative Ability of Assays

To assess the specificity of the multiplex one-step real-time qRT-PCR, the cross-reactivity of the monoplex primers/probe was examined first using all the *in vitro* transcribed viral RNA standards with the concentration of 10^6^ copies/µL, RNA extracts from viral isolates and human serum (140 µL of each sample producing 40 µL RNA extract) as templates. According to the criteria of qualitative determination in this study, the detection results of all the samples were determined. For testing of synthetic RNA standards, no cross-amplification reaction for any other virus was observed. All of the specific reactions had high positive fluorescence signals, and mean CTs were in the range of 15.22–20.13 (Table 2). In addition, there was also no significant non-specific amplification plots obtained in the testing of viral RNA extracts and healthy human sera (Table 3). The specificities of these 28 qRT-PCR assays were suggested to be 100% at a cut-off C_t_ value ≤35. It was indicated that the specificity of the developed one-step real-time qRT-PCR assay is considered satisfactory, and the primers/probe sets will be applicable for the multiplex assays.

**Table 2:** Specificity analysis using in vitro transcribed viral RNAs.

Assay	In vitro transcribed target viral RNA (5×10^6^ copies/PCR)																											
	HTNV	SEOV	PUUV	DOBV	TULV	BCCV	ANDV	SINV	CCHFV	RVFV	SFTSV	HLV	OHFV	KFDV	DENV-2	YFV	MARV	ZEBOV	SEBOV	CEBOV	JUNV	MACV	GTOV	SABV	CHAV	LASV	LUJV	BASV
**HTNV**	**20.13**	–	–	–	–	–	–	–	–	–	–	–	–	–	–	–	–	–	–	–	–	–	–	–	–	–	–	–
**SEOV**	–	**17.69**	–	–	–	–	–	–	–	–	–	–	–	–	–	–	–	–	–	–	–	–	–	–	–	–	–	–
**PUUV**	–	–	**18.25**	–	–	–	–	–	–	–	–	–	–	–	–	–	–	–	–	–	–	–	–	–	–	–	–	–
**DOBV**	–	–	–	**17.53**	–	–	–	–	–	–	–	–	–	–	–	–	–	–	–	–	–	–	–	–	–	–	–	–
**TULV**	–	–	–	–	**19.51**	–	–	–	–	–	–	–	–	–	–	–	–	–	–	–	–	–	–	–	–	–	–	–
**BCCV**	–	–	–	–	–	**17.67**	–	–	–	–	–	–	–	–	–	–	–	–	–	–	–	–	–	–	–	–	–	–
**ANDV/SINV**	–	–	–	–	–	–	**18.87**	**18.92**	–	–	–	–	–	–	–	–	–	–	–	–	–	–	–	–	–	–	–	–
**CCHFV**	–	–	–	–	–	–	–	–	**18.31**	–	–	–	–	–	–	–	–	–	–	–	–	–	–	–	–	–	–	–
**RVFV**	–	–	–	–	–	–	–	–	–	**19.01**	–	–	–	–	–	–	–	–	–	–	–	–	–	–	–	–	–	–
**SFTSV**	–	–	–	–	–	–	–	–	–	–	**18.46**	–	–	–	–	–	–	–	–	–	–	–	–	–	–	–	–	–
**HLV**	–	–	–	–	–	–	–	–	–	–	–	**17.58**	–	–	–	–	–	–	–	–	–	–	–	–	–	–	–	–
**OHFV**	–	–	–	–	–	–	–	–	–	–	–	–	**18.11**	–	–	–	–	–	–	–	–	–	–	–	–	–	–	–
**KFDV**	–	–	–	–	–	–	–	–	–	–	–	–	–	**18.62**	–	–	–	–	–	–	–	–	–	–	–	–	–	–
**DENV**	–	–	–	–	–	–	–	–	–	–	–	–	–	–	**18.55**	–	–	–	–	–	–	–	–	–	–	–	–	–
**YFV**	–	–	–	–	–	–	–	–	–	–	–	–	–	–	–	**17.65**	–	–	–	–	–	–	–	–	–	–	–	–
**MARV**	–	–	–	–	–	–	–	–	–	–	–	–	–	–	–	–	**17.39**	–	–	–	–	–	–	–	–	–	–	–
**ZEBOV**	–	–	–	–	–	–	–	–	–	–	–	–	–	–	–	–	–	**18.93**	–	–	–	–	–	–	–	–	–	–
**SEBOV**	–	–	–	–	–	–	–	–	–	–	–	–	–	–	–	–	–	–	**17.52**	–	–	–	–	–	–	–	–	–
**CEBOV**	–	–	–	–	–	–	–	–	–	–	–	–	–	–	–	–	–	–	–	**17.69**	–	–	–	–	–	–	–	–
**JUNV**	–	–	–	–	–	–	–	–	–	–	–	–	–	–	–	–	–	–	–	–	**18.82**	–	–	–	–	–	–	–
**MACV**	–	–	–	–	–	–	–	–	–	–	–	–	–	–	–	–	–	–	–	–	–	**17.25**	–	–	–	–	–	–
**GTOV**	–	–	–	–	–	–	–	–	–	–	–	–	–	–	–	–	–	–	–	–	–	–	**17.92**	–	–	–	–	–
**SABV**	–	–	–	–	–	–	–	–	–	–	–	–	–	–	–	–	–	–	–	–	–	–	–	**17.83**	–	–	–	–
**CHAV**	–	–	–	–	–	–	–	–	–	–	–	–	–	–	–	–	–	–	–	–	–	–	–	–	**18.32**	–	–	–
**LASV**	–	–	–	–	–	–	–	–	–	–	–	–	–	–	–	–	–	–	–	–	–	–	–	–	–	**18.13**	–	–
**LUJV**	–	–	–	–	–	–	–	–	–	–	–	–	–	–	–	–	–	–	–	–	–	–	–	–	–	–	**17.92**	–
**BASV**	–	–	–	–	–	–	–	–	–	–	–	–	–	–	–	–	–	–	–	–	–	–	–	–	–	–	–	**18.9**

The number indicates Ct value determined from three replicates;

The minus represents a negative detection;

**Table 3:** Specificity analysis using viral isolates and healthy human sera.

Assay	Viral isolates						Healthy human serum (positive/tested)
	HTNV	SEOV	SFTSV	DENV1–4*	YFV	CHIKV	
**HTNV**	**19.07**	–	–	–	–	–	0/200
**SEOV**	–	**17.33**	–	–	–	–	0/200
**PUUV**	–	–	–	–	–	–	0/200
**DOBV**	–	–	–	–	–	–	0/200
**TULV**	–	–	–	–	–	–	0/200
**BCCV**	–	–	–	–	–	–	0/200
**ANDV/SINV**	–	–	–	–	–	–	0/200
**CCHFV**	–	–	–	–	–	–	0/200
**RVFV**	–	–	–	–	–	–	0/200
**SFTSV**	–	–	**16.28**	–	–	–	0/200
**HLV**	–	–	–	–	–	–	0/200
**OHFV**	–	–	–	–	–	–	0/200
**KFDV**	–	–	–	–	–	–	0/200
**DENV**	–	–	–	**16.47, 18.12, 19.07, 15.13**	–	–	0/200
**YFV**	–	–	–	–	**15.22**	–	0/200
**MARV**	–	–	–	–	–	–	0/200
**ZEBOV**	–	–	–	–	–	–	0/200
**SEBOV**	–	–	–	–	–	–	0/200
**CEBOV**	–	–	–	–	–	–	0/200
**JUNV**	–	–	–	–	–	–	0/200
**MACV**	–	–	–	–	–	–	0/200
**GTOV**	–	–	–	–	–	–	0/200
**SABV**	–	–	–	–	–	–	0/200
**CHAV**	–	–	–	–	–	–	0/200
**LASV**	–	–	–	–	–	–	0/200
**LUJV**	–	–	–	–	–	–	0/200
**BASV**	–	–	–	–	–	–	0/200

The number indicates Ct value determined from three replicates;

The minus represents a negative detection;

*DENV1-4, 4 types of dengue virus, viral strains of Hawaii, New Guinea, H87 and H241 were used.

### Sensitivities and Detection Limits of Assays

To evaluate sensitivity, 10-fold serial dilutions of RNA standards (from 10^1^ to 10^8^ copies/µL) were used to estimate the detection limits of viral RNA copy load for the developed one-step real-time qRT-PCR assay. The amplification efficiencies of the monoplex assays for the 28 HFVs were all above 90%. The standard curves showed a high correlation coefficient, R^2^>0.99, for all the viruses detections (Supplementary Figure S1). The potential limits of detection (LODs) of these assays were determined to be at a range from 30 to 160 RNA copies/PCR (Table 4).

**Figure 1:**
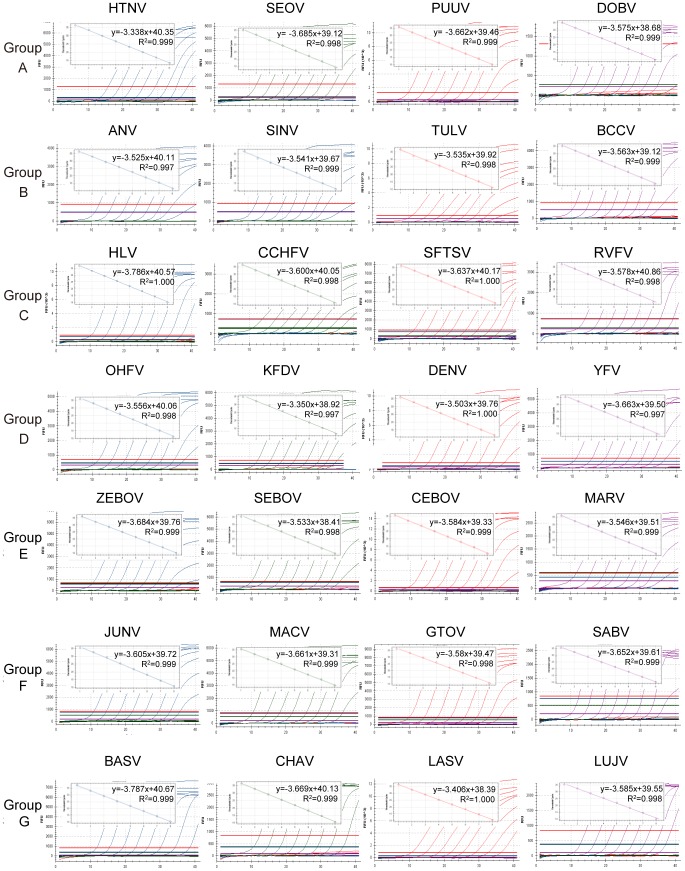
Amplification plots and standard curves of multiplex one-step real-time TaqMan RT-PCR assays. The multiplex one-step real-time TaqMan RT-PCR assays were tested using synthesized in vitro target viral RNA transcripts ranging from 10^1^ to 10^8^ copies/µL. A PCR baseline subtractive curve fit view of the data is shown with relative fluorescence units (RFUs) plotted against cycle numbers. Standard curves generated from the Ct values obtained against known concentrations, the coefficient of determination (R^2^) and slope of the regression curve for each assay are indicated.

**Table 4:** Detection limits of multiplex one-step real time qRT-PCR assays.

Group	Detected Viruses	Limits of detection (SEM) (Copies/PCR)			
		Monoplex assays		Multiplex assays	
**A**	**HTNV**	100.1	(19.1)	174.3	(22.3)
	**SEOV**	48.2	(8.1)	56.7	(11.5)
	**PUUV**	85.7	(14.9)	94.1	(10.6)
	**DOBV**	52.9	(6.1)	55.7	(4.7)
**B**	**TULV**	143.9	(14.3)	143.5	(17.6)
	**BCCV**	64.9	(12.7)	65.5	(10.5)
	**ANDV**	103.7	(18.3)	148.5	(14.6)
	**SINV**	86.3	(22.8)	124.3	(14.9)
**C**	**CCHFV**	133.5	(10.2)	114.4	(12.5)
	**RVFV**	155.7	(24.9)	215.6	(3.8)
	**SFTSV**	94.3	(18.5)	134.8	(2.5)
	**HLV**	103.5	(9.3)	146.0	(23.3)
**D**	**OHFV**	78.1	(14.8)	107.1	(23.2)
	**KFDV**	53.0	(16.8)	73.6	(1.2)
	**DENV**	114.2	(15.4)	124.8	(13.6)
	**YFV**	31.2	(24.9)	66.4	(25.2)
**E**	**MARV**	56.5	(3.1)	70.8	(19.6)
	**ZEBOV**	116.9	(9.4)	110.4	(14.5)
	**SEBOV**	46.1	(5.2)	46.5	(2.6)
	**CEBOV**	58.4	(7.4)	64.8	(14.1)
**F**	**JUNV**	105.6	(13.5)	116.5	(18.4)
	**MACV**	45.9	(9.6)	63.5	(14.0)
	**GTOV**	69.1	(16.4)	86.3	(11.8)
	**SABV**	68.0	(10.9)	82.3	(15.9)
**G**	**CHAV**	84.2	(5.8)	115.6	(18.9)
	**LASV**	53.2	(6.3)	50.0	(5.0)
	**LUJV**	62.2	(20.9)	75.8	(15.0)
	**BASV**	137.8	(23.2)	149.7	(7.4)

The synthesized RNA standards were then used for the multiplex assay testing, and standard curves of detections for each virus RNA transcripts were also constructed and showed high correlation coefficient, R^2^>0.99 (Figure 1). The results showed that the multiplex one-step real-time qRT-PCR assays could detect all the listed HFVs confirmed positive samples and no cross-reaction with the other examined RNA viruses was observed. In most multiplex assays (26 out of 28 virus detections), the LODs were at a range from 45 to 150 RNA copies/PCR, which was similar to that in the monoplex assays (Table 4). Besides, HTNV and RVFV detection assays showed a little lower sensitivity with the LODs of 174.3 and 215.6 copies/PCR, respectively. The analysis of the LOD indicated that the strategy of multiplex detection ensures the sensitivity of the assay system.

### Reproducibility of Multiplex One-step Real-time qRT-PCR Assay

**Figure 2:**
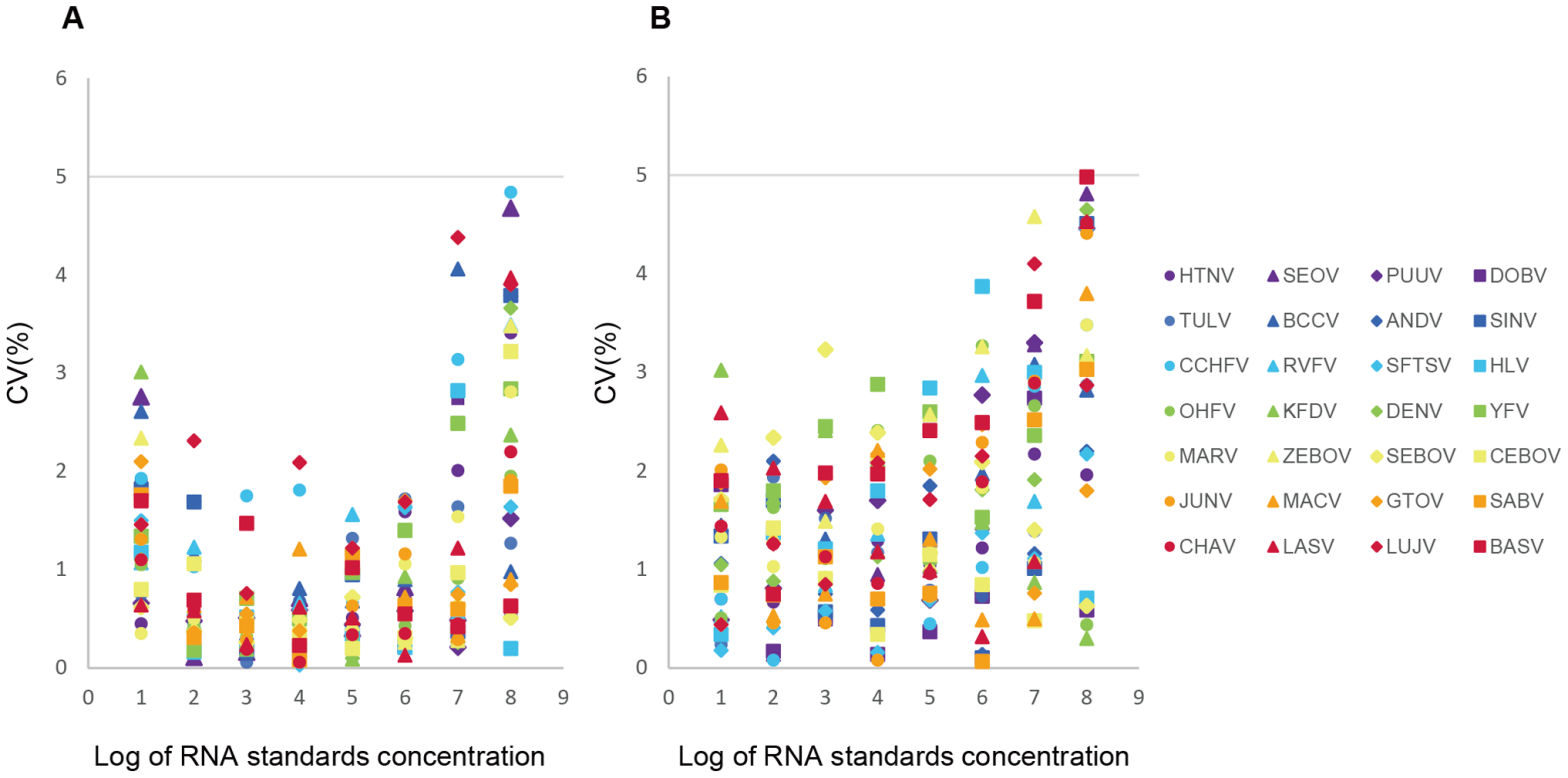
Coefficients of variation of Ct values in the multiplex one-step real-time RT-PCR assays. The multiplex one-step real-time RT-PCR assays were performed in three independent experiments of replicates. The Coefficients of variation (CV) of Ct values were calculated in both intra-assays (A) and inter-assays (B), and showed all less than 5%.

To assess the reproducibility of the multiplex one-step real-time qRT-PCR assay, mean C_T_ values were calculated at a serial dilution of viral RNA transcript standards (from 10^1^ to 10^8^ copies/µL), and the variations within and between runs in the linear range of the assays were statistically analyzed (Supplementary Table S3). The coefficients of variation (CVs) of C_T_ values were all less than 5% with 0.03–4.84% for intra-assays and 0.07–4.98% for inter-assays (Figure 2), suggesting that the developed multiplex one-step real-time qRT-PCR assay is reproducible.

### Evaluation using Clinical Specimens

To evaluate sensitivity and specificity of the multiplex one-step real-time qRT-PCR assays on clinical specimens, healthy human sera and sera from the respective patients infected with individual viruses were collected, in which HFRS patients, SFTS patients and Dengue fever patients were included. Three related groups of multiplex qRT-PCR assays, including Group A, Group C and Group D. were performed for test the diagnostic specificity and sensitivity in comparison with monoplex qRT-PCR assays. For the tested sera from 11 HFRS patients infected with HTNV and 48 SFTS patients infected with SFTSV, the assay sensitivity was 100% with all tested samples detected HTNV positive (11/11) in Group A or SFTSV positive (48/48) in Group C respectively, and the rest viruses in Group A (0/11) or C (0/48) were detected negative for above collected clinical samples (Table 5). The test of 53 sera collected from Dengue fever patients showed 96.2% sensitivity (51 out of 53 detected DENV positive) with only two negative samples (Table 5). There were no false positive results observed in the unrelated patients sera and healthy human sera, suggesting 100% specificity in all the three tested groups of multiplex assays (Table 5).

**Table 5:** Evaluation of the multiplex real-time qRT-PCR assays using clinical specimens.

Group	Detected Viruses	Patients sera* (positive/tested)			Healthy human sera (positive/tested)
		HFRS	SFTS	Dengue fever	
**A**	**HTNV**	**11/11**	0/48	0/53	0/100
	**SEOV**	0/11	0/48	0/53	0/100
	**PUUV**	0/11	0/48	0/53	0/100
	**DOBV**	0/11	0/48	0/53	0/100
**C**	**CCHFV**	0/11	0/48	0/53	0/100
	**RVFV**	0/11	0/48	0/53	0/100
	**SFTSV**	0/11	**48/48**	0/53	0/100
	**HLV**	0/11	0/48	0/53	0/100
**D**	**OHFV**	0/11	0/48	0/53	0/100
	**KFDV**	0/11	0/48	0/53	0/100
	**DENV**	0/11	0/48	**51/53**	0/100
	**YFV**	0/11	0/48	0/53	0/100

*Patient types were confirmed by sera detection using the corresponding monoplex assays.

## Discussion

VHFs are often fatal in spite of modern intensive care, especially some HFVs can be transmitted from human to human and consequently threat to cause epidemics with high mortality rates [21], [22]. Due to the unspecific clinical characters at the early phase of VHFs, a rapid and reliable laboratory testing appeared to be of utmost importance in diagnosis. Molecular assays based on RT-PCR have been successfully applied in the diagnosis of many types of VHFs [16]–[20]. However, most of these published methods only covered part of vial pathogens that caused VHFs. In this study, a multiplex quantitative real-time RT-PCR assay for detection of 28 HFVs which could be carried out in the same 96 wells plate was developed and evaluated. The assay sensitivity and specificity for diagnosis of HTNV, SFTSV and Dengue fever virus infection in patient sera were reliable and desirable. The assay system permits to use a universal reactive condition simultaneously to detect nearly all the hemorrhagic fever viral pathogens. Primers and probes were designed based on alignments of all possible representative viral genomic sequences in recent updated GenBank database, and optimized to react effectively under the same thermal cycling condition, which makes it possible to group these assays as required for broad-range multiplex screening of viral pathogens. It was difficult to obtain specific primers and probes to distinguish ANDV and SINV, thus these two viruses had to share the same primers/probes set to ensure that ANDV and SINV could be detected successfully and rapidly in the established universal system, and other detection methods could be used for further identification. For this point, the system we established in this study still remains to get improvement.

All the assays showed standard curves with high amplification efficiencies and strong linear correlations. The specificity and the reproducibility of the assays were demonstrated and the sensitivity of the systems was acceptable. No significant non-specific amplification was observed among the testing of 28 *in vitro* transcribed viral RNAs (Table 2), RNA extracts of Viruses isolates and 200 healthy human sera (Table 3), which suggested the high specificity of the primers/probe sets. In the multiplex assays testing, there was also no cross-amplification found in RNA transcripts of other viruses, indicating that the developed multiplex one-step real-time qRT-PCR assays were reliable in specificity. The CV of Ct values were all less than 5% in each dilution of synthesized viral RNAs for both intra-assays and inter-assays, suggesting that the multiplex assays were of good reproducibility (Figure 2). The LODs of these assays were determined in terms of viral RNA copy numbers, ranged from 30 to 160 RNA copies/PCR in the monoplex assays. In the followed multiplex assays, the main range of LODs was from 45 to 150 RNA copies/PCR, which covered more than 90% detected HFVs (Table 4). For HTNV detection assay, the LOD were influenced slightly by viral family based grouping of primers and probes into 4-plex reactions. It resulted in a lower sensitivity of HTNV detection in the multiplex testing with the LOD of 174.3 copies/PCR (Table 4). Another LOD beyond the main range was observed in the RVFV detection (215.6 copies/PCR). It may be, to some extent, due to the relative higher LOD of RVFV detection (155.7 copies/PCR) in the corresponding monoplex assay (Table 4). However, the overall of the sensitivities of multiplex assays was satisfactory, which made it possible to screen VHFs pathogens in one or two steps without requiring of large amount of clinical samples.

So far, most HFVs occurred in limited areas in the world, thus it is difficult to collect clinical samples of all of these 28 viral infections for assays evaluation and validation. The assays developed here were evaluated mostly based on chemically synthesized viral RNA standards, isolated viral RNAs from HFV strains (HTNV, SEOV, SFTSV, DENV 1–4 types, YFV and CHIKV) and healthy donor sera as negative control. It still remains the limitations of the assays to evaluate the specificities and sensitivities of the clinical diagnostic for those viruses included in this study because of the lack of clinical specimens. However, It is noteworthy that the LOD was determined by the linearity with statistically proved, resulting in representing the minimal number of copies that will be detected in 100% of the assays. The LODs and standard curves may be extended for quantitative analysis of clinical samples that are from infections caused by hemorrhagic fever viruses. Furthermore, evaluation with clinical samples of patients from three of hemorrhagic fever diseases, including HFRS caused by HTNV, SFTS caused by SFTSV and Dengue fever caused by DENV (Table 5) showed the reliable specificities and sensitivities for laboratory detection of the infections with these viruses and provided potential use for clinical diagnosis.

In conclusion, the comprehensive multiplex one-step real-time TaqMan qRT-PCR assays for rapid detection of 28 HFVs was established and evaluated in this study, which nearly covered all the hemorrhagic fever viruses. The developed multiplex one-step real-time qRT-PCR assay was tested using different simulate samples and showed excellent parameters in the followed statistical analysis. Therefore, this assay proved to be specific, sensitive and, apparently, convenient for rapid and simultaneous identification in laboratory, and could be certainly extended to routine diagnosis and epidemiological detection of VHF infections.

## Materials and Methods

### Primers and Probes Design

Genomic sequences used in the study were all retrieved from the GenBank database of NCBI (http://www.ncbi.nlm.nih.gov/nuccore/), including the nucleotide sequences of the full genome of the HFVs in the families of *Flaviviridae* and *Filoviridae*, the S segments of the viruses in the families of *Bunyaviridae* and *Arenaviridae*, and the only one genomic sequence of BASV. The multiple alignments of genomic sequences were carried out respectively using Clustal W within the sequence editor package BioEdit (version 7.0.9) to identify conserved regions by visual inspection of the sequence alignments [23]. Primers and probe for each virus were designed using a Primer Express software (version 3.0, Applied Biosystems), and optimized using DNASTAR software by analysis of potentials for dimerization, cross-linking and secondary structures. The specificity of primer and probe sequences was further confirmed using primer–BLAST (NCBI). The probes were differently labeled with the fluorescent dyes, FAM, HEX, TEXAS RED or CY5. All oligonucleotides were synthesized by Shanghai Sangon Biotech Co., Ltd.

### Viruses and Samples

Viral isolates propagated in C6/36 or Vero cells, including HTNV (84Fli strain), SEOV (L99 strain), SFTSV (HB29 strain), DENV 1–4 types (Hawaii, New Guinea, H87 and H241 strains), YFV (17D strain) and Chikungunya fever virus (CHIKV, SD08Pan strain), were prepared. Human serum samples from healthy adult donors (n = 200) were assembled from samples library of Chinese National Institute for Viral Diseases Control and Prevention. Among them, CHIKV isolates and healthy human sera were used as negative control in all the tests, whereas the other viral isolates were implied as positive or negative control in the detection assays for different viruses. The human sera from HFRS patients (N = 11), SFTS patients (N = 48) and Dengue fever patients (N = 53) in the acute phase were from our laboratory collections, which were all confirmed by monoplex real-time qRT-PCR assays, and other specific detection methods (virus isolation or IgG detection).

### RNA Extraction

RNAs from sera and the culture supernatant of virus-infected cells were extracted from 140 µL of each sample using QIAamp Viral RNA Mini Kit (Qiagen) according to the manufacturer’s instructions, then eluted in 40 µL sterilized RNase free water and stored at −80°C before use.

### Preparation of Viral RNA Standards

Preparation of viral RNA standards was initiated with viral genomic DNA fragments obtained through chemical synthesis or RT-PCR amplification from viral isolates (HTNV, SEOV, SFTSV, DENV and YFV). The T7 promoter sequence was then introduced into the 5′ terminus (for positive-strand viruses) or 3′ terminus (for negative-strand viruses) of these DNA fragments via PCR amplification. The PCR products were purified using the Gel Extraction Kit (Qiagen) and used for *in vitro* transcription with a RiboMAX™ Large Scale RNA Production Systems-T7 (Promega). The synthetic RNA transcripts were purified by RNeasy Mini Kit (Qiagen), followed by concentration calculation using a NanoDrop ND-1000 spectrophotometer (NanoDrop Technologies) and analysis by 2% agarose gel electrophoresis, and then stored at −80°C. Dilutions of viral RNA standards ranging from 10^1^ to 10^8^ copies/µL were prepared by 10-fold serial dilution of RNA transcripts in sterilized RNase free water according to the concentration and length of each transcript.

### One-step Real-time qRT-PCR Assays

One-step real-time qRT-PCR reactions were performed using AgPath-ID™ one-step RT-PCR Kit (Applied Biosystems), and contained 12.5 µL of 2× RT-PCR buffer, 400 nM of each primer, 120 nM of each probe, 1 µL of Enzyme Mix and 5 µL of viral RNA transcripts or RNA extracts in a final volume of 25 µL. Total RNA extracted from Hela cells was used as negative control. Real-time qRT-PCR cycling was performed on Bio-Rad CFX96 system as follows: 50°C for 30 min, 95°C for 10 min, then 40 cycles of 15 s at 95°C and 45 s at 60°C. The fluorogenic signal emitted was collected at the end of annealing-extension step. A threshold was automatically set and the threshold cycle value (C_t_) was consequently determined. Three replicates of the assay within or between runs were performed. Multiplex assays were assembled by grouping the primers and probes according to the hosts/vectors or viral families (Supplementary Table S3), reaction conditions were as same as described above.

### Specificity, Sensitivity and Reproducibility

To assess the specificities of the developed one-step real-time qRT-PCR assays, each pair of primers and probe was tested in triplicate against all the other *in vitro* synthetic viral RNA transcripts with the concentration of 10^6^ copies/µL, RNA extracts of HTNV, SEOV, SFTSV, DENV, YFV and CHIKV, as well as serum RNA from a panel of 200 sera from human without VHFs.

To evaluate sensitivity of monoplex and multiplex one-step real-time qRT-PCR assays, each group of 10-fold serial dilutions of 28 *in vitro* synthetic target viral RNA transcripts, ranging from 10^1^ to 10^8^ copies/µL, were used as standard preparations to assess the detection limits of viral RNA copy load. Three replicates of the assay within or between runs were performed to assess the reproducibility, and the intra-assay and inter-assay variations over the linear range of the assays were statistically calculated.

### Statistical Analysis

Regression, reproducibility and the coefficient of variation (CV) of the mean C_t_ value for each standard concentration within and between individual PCR runs were statically calculated by using GraphPad Prism version 5.01 (GraphPad Software, San Diego, CA; http://www.graphpad.com/prism/Prism.htm) to evaluate linearity and determine the quantitative performance of each assay.

### Ethical Consideration

According to the medical research regulation of National Health and Family Planning Commission, China, all studies involved in human samples were reviewed and approved by the ethics committee of China Center for Disease Control and Prevention, which uses international guidelines to ensure confidentiality, anonymity, and informed consent. The written informed consent was agreed by the donors.

## Supporting Information

Figure S1 **Amplification plots and standard curves of monoplex one-step real-time TaqMan qRT-PCR assays.** The monoplex one-step real-time TaqMan RT-PCR assays were performed with synthesized in vitro target viral RNA transcripts ranging from 10^1^ to 10^8^ copies/µL to evaluate the specificity and the sensitivity of each primers/probe set. A PCR baseline subtractive curve fit view of the data is shown with relative fluorescence units (RFUs) plotted against cycle numbers. Standard curves generated from the Ct values obtained against known concentrations, the coefficient of determination (R^2^) and slope of the regression curve for each assay are indicated.
(PDF)

Table S1 **GenBank accession numbers of hemorrhagic fever viruses aligned in this study.**
(PDF)

Table S2 **Viral RNA standards prepared via in vitro transcription.**
(PDF)

Table S3 **Reproducibility analysis of multiplex one-step real-time RT-PCR assays.**
(PDF)
