# Supplementary material for: Comprehensive Multiplex One-Step Real-Time TaqMan qRT-PCR Assays for Detection and Quantification of Hemorrhagic Fever Viruses
Source: PLoS One. 2014 Apr 21;9(4):e95635. doi: 10.1371/journal.pone.0095635 (PMC3994070; doi:10.1371/journal.pone.0095635)
Supplement: Figure S1 — Amplification plots and standard curves of monoplex one-step real-time TaqMan qRT-PCR assays. The monoplex one-step real-time TaqMan RT-PCR assays were performed with synthesized in vitro target viral RNA transcripts ranging from 101 to 108 copies/µL to evaluate the specificity and the sensitivity of each primers/probe set. A PCR baseline subtractive curve fit view of the data is shown with relative fluorescence units (RFUs) plotted against cycle numbers. Standard curves generated from the Ct values obtained against known concentrations, the coefficient of determination (R2) and slope of the regression curve for each assay are indicated. (PDF) [file pone.0095635.s001.pdf]

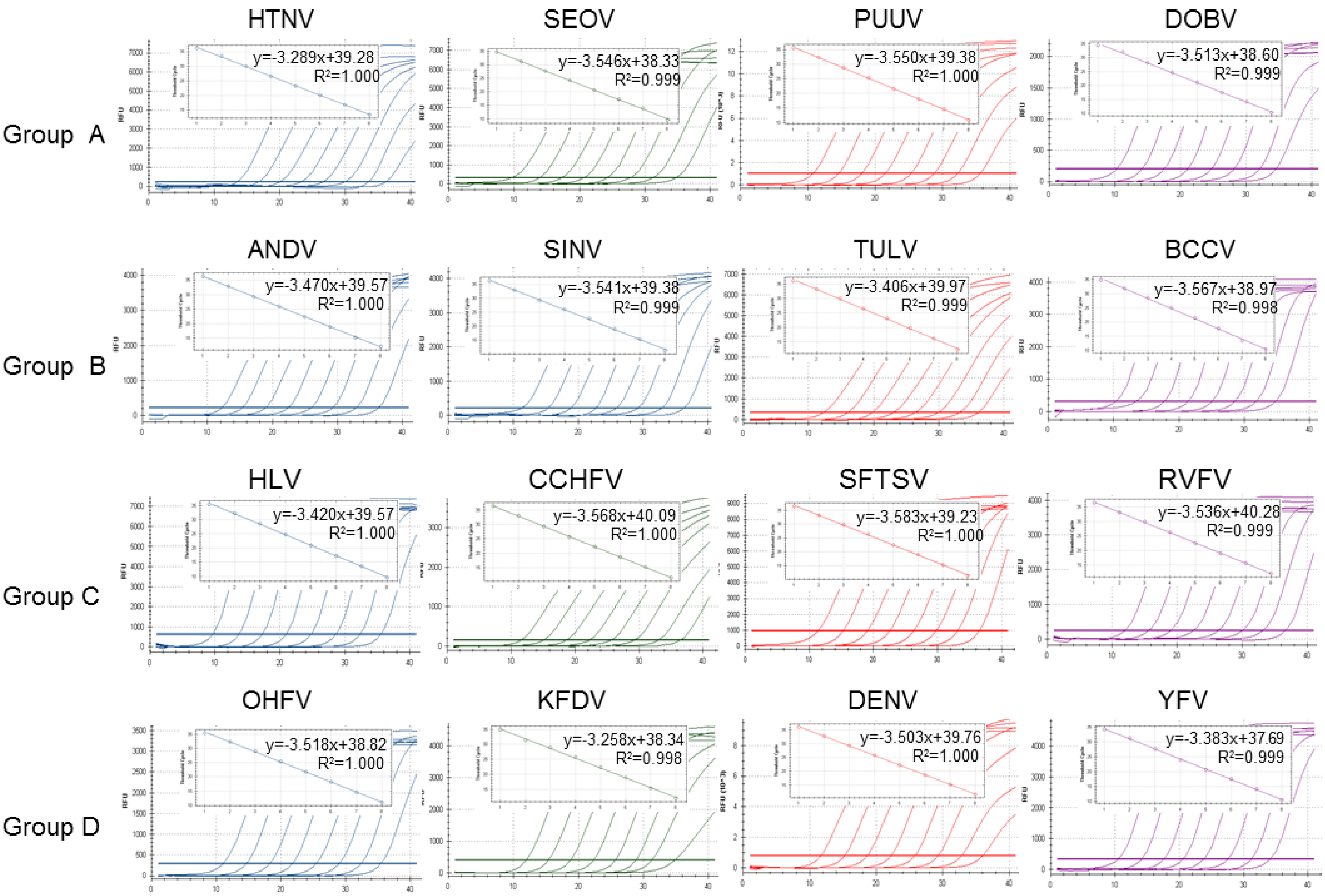
Group A
HTNV
y=-3.289x+39.28
R²=1.000
SEOV
y=-3.546x+38.33
R²=0.999
PUUV
y=-3.550x+39.38
R²=1.000
DOBV
y=-3.513x+38.60
R²=0.999
Group B
ANDV
y=-3.470x+39.57
R²=1.000
SINV
y=-3.541x+39.38
R²=0.999
TULV
y=-3.406x+39.97
R²=0.999
BCCV
y=-3.567x+38.97
R²=0.998
Group C
HLV
y=-3.420x+39.57
R²=1.000
CCHFV
y=-3.568x+40.09
R²=1.000
SFTSV
y=-3.583x+39.23
R²=1.000
RVFV
y=-3.536x+40.28
R²=0.999
Group D
OHFV
y=-3.518x+38.82
R²=1.000
KFDV
y=-3.258x+38.34
R²=0.998
DENV
y=-3.503x+39.76
R²=1.000
YFV
y=-3.383x+37.69
R²=0.999
RFU

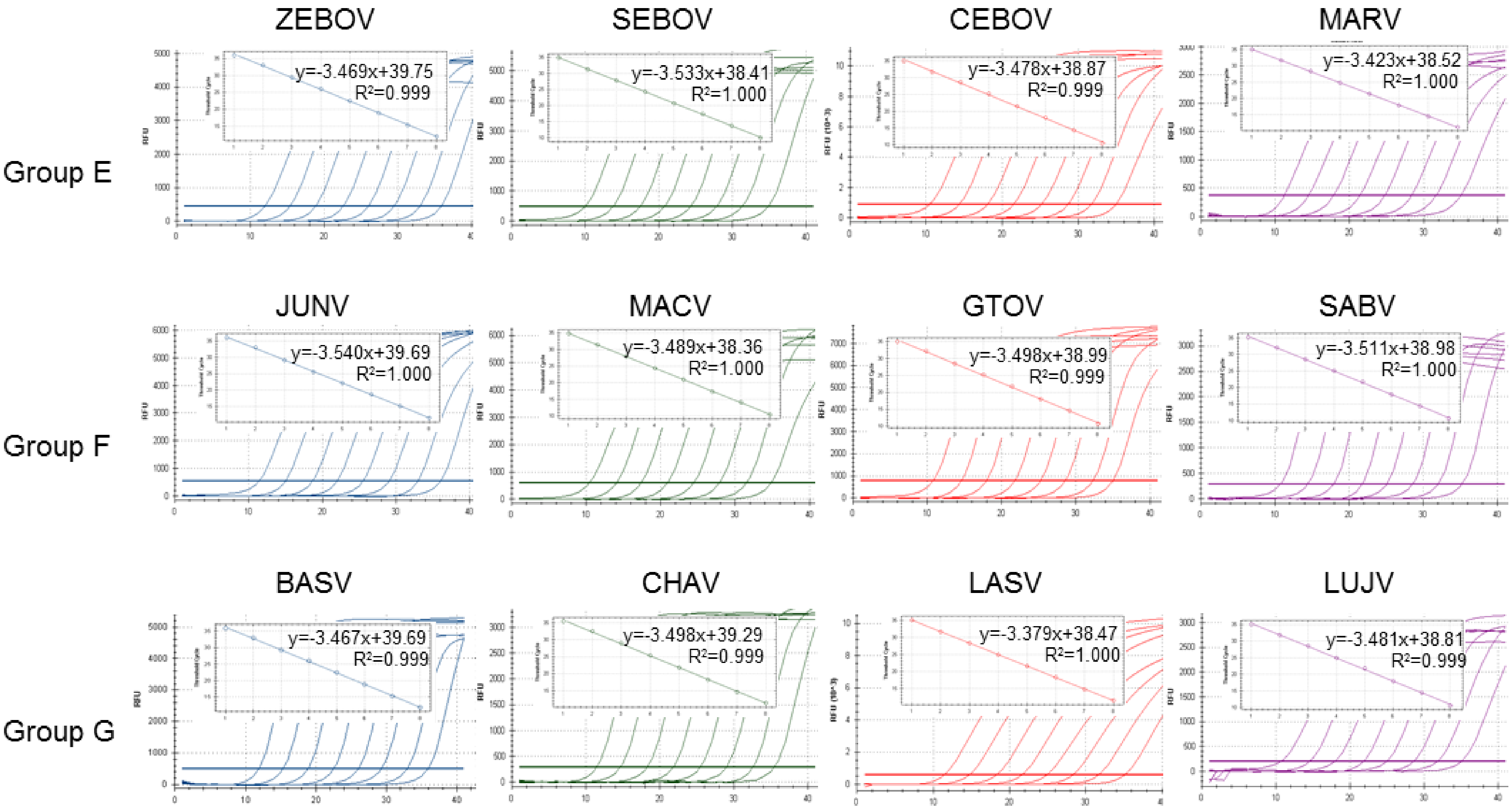

Figure S1. Amplification plots and standard curves of monoplex one-step real-time TaqMan RT-PCR assays.

The monoplex one-step real-time TaqMan RT-PCR assays were performed with synthesized in vitro target viral RNA transcripts ranging from 10^1 to 10^8 copies / μL to evaluate the specificity and the sensitivity of each primers/probe set. A PCR baseline subtractive curve fit view of the data is shown with relative fluorescence units (RFUs) plotted against cycle numbers. Standard curves generated from the Ct values obtained against known concentrations, the coefficient of determination (R^2) and slope of the regression curve for each assay are indicated.
