# Supplementary material for: Comprehensive Multiplex One-Step Real-Time TaqMan qRT-PCR Assays for Detection and Quantification of Hemorrhagic Fever Viruses
Source: PLoS One. 2014 Apr 21;9(4):e95635. doi: 10.1371/journal.pone.0095635 (PMC3994070; doi:10.1371/journal.pone.0095635)
Supplement: Table S1 — GenBank accession numbers of hemorrhagic fever viruses aligned in this study. (PDF) [file pone.0095635.s002.pdf]

Table S1. GenBank accession numbers of hemorrhagic fever virus sequences aligned in this study

Families	Genus	Species	GenBank accession numbers	Total numbers
**Bunyaviridae**	**Hantavirus**	**Hantaan virus**	NC_005218.1, AB027097.1, AB027101.1, AB027111.1, AB027523.1, AB127998.1, AB620031.1, AF288294.1, AF288296.1, AF288644.1, AF288646.1, AF288657.1, AF288659.1, AF321094.1, AF321095.1, AF366568.1, AF427318.1-AF427324.1, AY017064.1, AY748308.1, AY748309.1, AY839871.1, D25530.1, D25533.1, DQ658415.1, EF208929.1, EF595840.1, EF990904.1-EF990915.1, EU092218.1-EU092221.1, EU363808.1-EU363813.1, FJ753396.1, FJ753398.1, HQ611981.1, HQ834499.1-HQ834507.1, M14626.1, U37768.1, X95077.1	69
		**Seoul virus**	NC_005236.1, AB027522.1, AB618143.1, AB618144.1, AF184988.1, AF187082.1, AF288295.1, AF288299.1, AF288643.1, AF288651.1, AF288653.1, AF288655.1, AF329388.1-AF329390.1, AF406965.1, AF488708.1, AY006465.1, AY273791.1, AY605933.1, AY627049.2, AY750171.1, AY766368.1, EF192308.1, EF536376.1, FJ753400.1, FJ803201.1, FJ803202.1, FJ803205.1- FJ803208.1, FJ803210.1-FJ803217.1, GQ274944.1, GQ274945.1, GQ279379.1-GQ279395.1, GU361893.1, GU592932.1-GU592953.1, HQ611980.1	83
		**Puumala virus**	NC_005224.1, AB010730.1, AB010731.1, AB433843.2-AB433848.1, AF294652.1, AF367064.1-AF367071.1, AF442613.1, AJ238788.1-AJ238791.1, AJ277030.1-AJ277034.1, AJ277075.1, AJ277076.1, AJ278092.1, AJ278093.1, AJ314597.1-AJ314601.1, AJ888751.1, AJ888752.1, AM695638.1, AY526219.1, AY954722.2, AY954723.2, DQ016430.2-DQ016432.2, FN377821.1, FN377822.1, GQ339473.1-GQ339487.1, GU808824.1, GU808825.1, M32750.1, U14137.1, X61035.1, Z21497.1, Z30702.1-Z30708.1, Z46942.1, Z48586.1, Z84204.1	79

		**Dobrava virus**	NC_005233.1, AF442622.1, AF442623.1, AJ009773.1, AJ009775.1, AJ131672.1, AJ131673.1, AJ269549.1, AJ269550.1, AJ410615.1, AJ410619.1, AJ616854.1, AY168576.1, AY533118.2, AY533120.2, AY961615.1, AY961618.1, EU188449.1, EU188452.1, EU562989.1-EU562991.1, GQ205401.1-GQ205408.1, GU904027.1-GU904032.1, L41916.1	37
		**Tula virus**	NC_005227.2, AF017659.1, AF063892.1, AF063897.1, AF164093.1, AF164094.1, AF289819.1- AF289821.1, AF442621.1, AM945877.1, AJ223600.1, AJ223601.1, EU439949.1- EU439951.1, Y13979.1, Y13980.1, Z30941.1- Z30945.1, Z48573.1,Z48574.1, Z48741.1, Z49915.1, Z69991.1	28
		**Black creek canal virus**	L39949.1	1
		**Andes virus**	NC_003466.1, AF004660.1, AF291702.1, AF324902.1, AF325966.1, AF482713.1-AF482717.1, AY228237.1, AY267347.1, DQ345763.1, EF571895.1	14
		**Sin nombre virus**	NC_005216.1, AF281850.1, AF281851.1 L25784.1, L33683.1, L33816.1, L37904.1, U47135.1	8
	**Nairovirus**	**Crimean-Congo hemorrhagic fever virus**	NC_005302.1, AF354296.1, AF362080.1, AF428144.3, AF481799.1, AF481802.1, AF527810.1, AJ010648.1, AJ010649.1, AJ538196.1, AJ538198.1, AY029157.1, AY049083.2, AY223475.1, AY277672.1, AY277676.2, AY297691.2, AY297692.2, DQ076413.1, DQ076415.1, DQ076416.1, DQ133507.1, DQ144418.1, DQ206447.1, DQ211638.1-DQ211650.1, DQ217602.1, DQ227495.1, DQ227496.1, DQ446212.1-DQ446214.1, EF123122.1, EU727456.1, FJ562093.1, GQ337053.1, GQ862371.1, GQ862372.1, GU477489.1, GU477494.1, HM452305.1, M86624.1, M86625.1, U04958.1, U88410.1-U88416.1	62
	**Phlebovirus**	**Rift Valley fever virus**	NC_014395.1, DQ380143.1-DQ380181.1, EU312103.1-EU312147.1, EU574057.1-EU574087.1, EU709747.1, EU709748.1, X53771.1	119
		**Severe fever with**	NC_018137.1,HM745932.1, HM802204.1, HM802205.1, HQ141591.1, HQ141594.1,	47

		**thrombocytopenia syndrome virus**	HQ141597.1, HQ141600.1, HQ141603.1, HQ141606.1, HQ141609.1, HQ141612.1, HQ171191.1-HQ171194.1, HQ419239.1-HQ419244.1, HQ830165.1, HQ830168.1, HQ830171.1, JQ317171.1, JQ317174.1, JQ317177.1, JQ317180.1, JQ670932.1, JQ670933.1, JQ684873.1, JQ693001.1-JQ693013.1, JQ733562.1, JQ733565.1, JQ733568.1	
		**Heartland virus**	JX005842.1, JX005843.1	2
**Flaviviridae**	**Flavivirus**	**Omsk hemorrhagic fever virus**	NC_005062.1, AB507800.1, AY193805.1, AY323489.1, AY438626.1	5
		**Kyasanur forest disease virus**	NC_004355.1, AF331718.1, AY323490.1, DQ154114.1, EU480689.1, HM055369.1	6
		**Dengue virus**	**DENV1:** AF311957, AF311958, AF513110, EU482497, EU482500-EU482502, EU482509, EU482511, EU482512, EU482515, EU482516, EU482521, EU482525, EU482526, EU482533-EU482535, EU482538, EU482539, EU482567, EU482706, EU482800, EU482802, EU482803, EU482822, EU482823, EU596501, EU660390, EU660391, EU687247, EU848545, FJ024423, FJ024440, FJ024441, FJ024442, FJ024446, FJ024448, FJ024472, FJ024480, FJ024481, FJ205873, FJ205874, FJ410290, FJ432720, FJ461307, FJ461308, FJ461310, FJ461330, FJ461335, FJ461336, FJ461341, FJ639669, FJ639670, FJ639671, FJ639673,-FJ639678, FJ639680-FJ639684, FJ639686, FJ639688, FJ639692, FJ639796, FJ639797, FJ639802, FJ639812-FJ639814, FJ639824, FJ687432, FJ687433, FJ744701, FJ744702, FJ810415, FJ810419, FJ850068, FJ850069, FJ898391, FJ898423, FJ898424, FJ898430, FJ898431, FJ898433, FJ898437, FJ898448, FN429881-FN429883, FN429887, FN429889, FN429890, GQ199771, GQ199772, GQ199791, GQ199793, GQ199794, GQ199817-GQ199819, GQ199827-GQ199829, GQ199831-GQ199833, GQ199836-GQ199838, GQ199852-GQ199854, GQ199856-GQ199859, GQ199873, GQ199875, J461323, J639823<br>**DENV2:** NC_001474, AB122020-AB122024, AF489932, AY702034, AY702040,	326

			AY744147, AY858035, AY858036, DQ181797, DQ181798, DQ181803, DQ181804, DQ181806, EF051521, EF457904, EU056810, EU056811, EU056812, EU179857-EU179859, EU359009, EU482608, EU660415, EU677145, EU687212, EU687213, EU687217, EU687220, EU687225, EU687232, EU687241-EU687243, EU687246, EU726767, EU726775, EU781135, FJ024475, FJ024477, FJ182012, FJ226066, FJ390389, FJ410259, FJ410288, FJ432726, FJ461311, FJ639700, FJ639705, FJ639706, FJ639711, FJ639717, FJ639718, FJ639783, FJ639822, FJ810412, FJ850067, FJ850072, FJ850074, FJ850076, FJ850078, FJ850082, FJ850085, FJ850088, FJ850108, FJ850112, FJ906962, FM210202, FM210204, FM210206-FM210213, FM210216-FM2102123, FM210231-FM210234, FM210236-FM210244, FN429891, FN429892, FN429895, GQ199869, GQ199874, GQ199890, GQ199892, GQ199893, GQ199895-GQ199898, GQ199901, GQ252676, GQ252677, M20558, M29095, MD1515<br>**DENV3:** NC_001475, AY099337, AY766104, AY770511, DQ863638, EU529699, EU660420, EU854292, FJ182013, FJ182041, FJ898441-FJ898445, FJ898455-FJ898459, FJ898462-FJ898464, FJ898468, FJ898471, FJ898472, FJ898474, FN429897-FN429900, FN429904, FN429907, FN429909, FN429911, FN429913, GQ199889, GQ199891, GQ252674, GQ252678, M93130<br>**DENV4:** AY947539, EU854295-EU854297,EU854299-EU854301, FJ024424, FJ024476, FJ182016, FJ182017, FJ882590-FJ882592, FJ882595-FJ882601, FN429919-FN429922, FN429924-FN429926, GQ199876-GQ199882, GQ199884, GQ252675, MY0327498, MY95328	
		**Yellow fever virus**	NC_002031.1, AF094612.1, AY572535.1, AY603338.1, AY640589.1, AY968064.1, AY968065.1, DQ100292.1, DQ118157.1, DQ235229.1, FJ654700.1, GQ379162.1, GQ379163.1, JN620362.1, JN628279.1- JN628281.1, U17066.1, U17067.1, U21055.1, U21056.1, U54798.1, X03700.1, X15062.1	24
**Filoviridae**	**Filovirus**	**Marburg virus**	NC_001608.3, AY358025.2, AY430365.1, AY430366.1, DQ217792.1, EF446131.1,	9

			EF446132.1, Z12132.1, Z29337.1	
		**Ebola virus**	**ZEBOV:** NC_002549.1, AF086833.2, AF272001.1, AF499101.1, AY142960.1, AY354458.1, EU224440.2, L11365.1, **SEBOV:** NC_006432.1, AY729654.1, EU338380.1, FJ968794.1 **CEBOV:** NC_014372.1, FJ217162.1	14
**Arenaviridae**	**Arenavirus**	**Junin virus**	NC_005081.1, AY358023.2, AY619641.1, AY746353.1, D10072.2, DQ272266.3, DQ531486.1, DQ531488.1, DQ854730.1- DQ854739.1, FJ805378.1, FJ805380.1, FJ969442.1, GQ121040.1, U70799.1- U70804.1,	28
		**Machupo virus**	NC_005078.1, AF485260.1, AY129248.1, AY619643.1, AY619645.1, AY624355.1, AY924202.1- AY924208.1, FJ696411.1- FJ696415.1	18
		**Guanarito virus**	NC_005077.1, AF485258.1, AY129247.1, AY497548.1, AY572557.1- AY572561.1, AY573922.1, AY576604.1	11
		**Sabia virus**	NC_006317.1, U41071.1	2
		**Chapare virus**	NC_010562.1, EU260463.1	2
		**Lassa virus**	NC_004296.1, AY179173.1, AY628201.1, AY628203.1, AY628205.1- AY628208.1, GU481068.1, GU481070.1, GU481072.1, GU481074.1, GU481076.1, GU481078.1, GU830839.1, HQ688672.1, HQ688673.1, J04324.1, X52400.1	19
		**Lujo virus**	NC_012776.1, FJ952384.1	2
**Rhabdoviridae**	**Rhabdovirus**	**Bas-Congo virus**	JX297815.1	1
