# Supplementary material for: Comprehensive Multiplex One-Step Real-Time TaqMan qRT-PCR Assays for Detection and Quantification of Hemorrhagic Fever Viruses
Source: PLoS One. 2014 Apr 21;9(4):e95635. doi: 10.1371/journal.pone.0095635 (PMC3994070; doi:10.1371/journal.pone.0095635)
Supplement: Table S2 — Viral RNA standards prepared via in vitro transcription. (PDF) [file pone.0095635.s003.pdf]

Table S2. Viral RNA standards prepared via *in vitro* transcription

Virus	Source	GenBank accession number of the referenced sequence	Length (nt)	Concentration (ng/µL)	Copy number (copies/µL)
Hantaan virus	Virus isolate	AY017064.1	1290	820	1.125E12
Seoul virus	Virus isolate	AF288299.1	1290	600	8.235E11
Puumala virus	Chemical synthesis	NC_005224	1302	814	1.107E12
Dobrava virus	Chemical synthesis	NC_005233	1290	792	1.087E12
Tula virus	Chemical synthesis	NC_005227	1290	837	1.148E12
Black creek canal virus	Chemical synthesis	L39949	1394	791	1.004E12
Andes virus	Chemical synthesis	NC_003466	1287	1026	1.411E12
Sin nombre virus	Chemical synthesis	NC_005216.1	1287	418	5.749E11
Crimean-Congo hemorrhagic fever virus	Chemical synthesis	NC_005302	1449	801	9.784E11
Rift Valley fever virus	Chemical synthesis	NC_014395	738	867	2.079E12
Severe fever with thrombocytopenia syndrome virus	Virus isolate	NC_018137.1	738	1120	2.686E12
Heartland virus	Chemical synthesis	JX005842.1	738	951	2.280E12
Omsk hemorrhagic fever virus	Chemical synthesis	NC_005062	966	820	1.502E12
Kyasanur forest disease virus	Chemical synthesis	NC_004355	2709	381	2.489E11
Dengue virus type 2	Virus isolate	AF204178.1	967	856	1.567E12
Yellow fever virus	Virus isolate	X03700.1	1011	896	1.569E12
Marburg virus	Chemical synthesis	NC_001608	2796	737	4.666E11
Ebola Zaire virus	Chemical synthesis	NC_002549	2965	646	3.856E11
Ebola Sudan virus	Chemical synthesis	NC_006432	2965	432	2.579E11
Ebola Cote d'Ivoire virus	Chemical synthesis	NC_014372	2965	695	4.149E11
Junin virus	Chemical synthesis	NC_005081	714	200	4.958E11
Machupo virus	Chemical synthesis	NC_005078	1491	729	8.654E11
Guanarito virus	Chemical synthesis	NC_005077	1440	322	3.958E11
Sabia virus	Chemical synthesis	NC_006317	1467	211	2.546E11
Chapare virus	Chemical synthesis	NC_010562	1455	722	8.783E11
Lassa virus	Chemical synthesis	NC_004296	1476	712	8.538E11
Lujo virus	Chemical synthesis	NC_012776	1365	315	4.085E11
Bas-Congo virus	Chemical synthesis	JX297815	1224	1388	1.912E12
