# Supplementary material for: Comprehensive Multiplex One-Step Real-Time TaqMan qRT-PCR Assays for Detection and Quantification of Hemorrhagic Fever Viruses
Source: PLoS One. 2014 Apr 21;9(4):e95635. doi: 10.1371/journal.pone.0095635 (PMC3994070; doi:10.1371/journal.pone.0095635)
Supplement: Table S3 — Reproducibility analysis of multiplex one-step real-time RT-PCR assays. (PDF) [file pone.0095635.s004.pdf]

Table S3. Reproducibility analysis of multiplex one-step real-time RT-PCR assays

Assay	RNA transcripts	RNA transcripts concentration	Mean Ct value	Intra-assay CV (%)	Inter-assay CV (%)
Group A	HTNV	10^8copies/μl	13.54	3.41	1.96
		10^7copies/μl	16.70	2.01	2.17
		10^6copies/μl	20.04	1.59	1.22
		10^5copies/μl	23.24	0.51	1.05
		10^4copies/μl	26.57	0.30	1.29
		10^3copies/μl	30.23	0.73	0.53
		10^2copies/μl	33.53	0.56	0.67
		10^1copies/μl	36.29	0.45	0.70
	SEOV	10^8copies/μl	10.72	4.68	4.81
		10^7copies/μl	14.37	0.56	3.28
		10^6copies/μl	17.78	0.82	1.47
		10^5copies/μl	21.33	0.40	1.30
		10^4copies/μl	24.71	0.71	0.95
		10^3copies/μl	28.30	0.16	0.50
		10^2copies/μl	31.79	0.11	0.14
		10^1copies/μl	35.21	2.76	1.69
	PUUV	10^8copies/μl	10.82	1.52	4.46
		10^7copies/μl	14.68	0.21	3.30
		10^6copies/μl	18.11	0.58	2.77
		10^5copies/μl	21.47	0.44	0.69
		10^4copies/μl	25.44	0.59	1.70
		10^3copies/μl	29.08	0.51	1.60
		10^2copies/μl	32.48	0.48	0.81
		10^1copies/μl	35.96	0.66	0.49
	DOBV	10^8copies/μl	10.28	0.59	0.59
		10^7copies/μl	13.94	2.74	2.74
		10^6copies/μl	17.60	0.73	0.73
		10^5copies/μl	21.17	0.37	0.37
		10^4copies/μl	24.71	0.14	0.14
		10^3copies/μl	28.26	0.52	0.52
		10^2copies/μl	32.01	0.17	0.17
		10^1copies/μl	35.07	1.86	1.86
Group B	TULV	10^8copies/μl	12.17	1.27	2.87
		10^7copies/μl	15.78	1.64	1.39
		10^6copies/μl	19.4	1.72	0.73
		10^5copies/μl	22.75	1.32	0.79
		10^4copies/μl	26.34	0.68	1.17
		10^3copies/μl	29.87	0.06	1.52
		10^2copies/μl	33.28	0.45	1.94
		10^1copies/μl	36.39	0.75	0.24
	BCCV	10^8copies/μl	10.17	0.98	2.82
		10^7copies/μl	14.05	4.06	3.08
		10^6copies/μl	17.88	0.90	1.97
		10^5copies/μl	21.41	0.68	1.03
		10^4copies/μl	25.18	0.81	2.15
		10^3copies/μl	28.54	0.35	1.31
		10^2copies/μl	32.13	1.15	0.79
		10^1copies/μl	35.80	2.61	1.45
	ANDV	10^8copies/μl	11.89	3.79	2.20
		10^7copies/μl	15.36	0.37	1.16
		10^6copies/μl	18.98	0.21	0.14
		10^5copies/μl	22.54	0.95	1.85
		10^4copies/μl	26.10	0.19	0.59
		10^3copies/μl	29.32	0.17	0.75
		10^2copies/μl	32.7	1.69	2.10
		10^1copies/μl	37.12	1.82	1.06

	SINV	10^8copies/μl	11.48	3.79	4.51
		10^7copies/μl	15.24	0.37	1.01
		10^6copies/μl	19.02	0.21	0.105
		10^5copies/μl	22.48	0.95	1.31
		10^4copies/μl	26.02	0.19	0.43
		10^3copies/μl	29.20	0.17	0.57
		10^2copies/μl	32.36	1.69	1.70
		10^1copies/μl	36.87	1.82	1.34
Group C	CCHFV	10^8copies/μl	10.80	4.84	3.48
		10^7copies/μl	14.87	3.14	2.86
		10^6copies/μl	18.25	0.25	1.02
		10^5copies/μl	21.97	0.66	0.45
		10^4copies/μl	25.64	1.81	2.07
		10^3copies/μl	29.25	1.75	0.86
		10^2copies/μl	32.76	1.03	0.08
		10^1copies/μl	36.64	1.93	0.70
	RVFV	10^8copies/μl	10.32	3.49	4.52
		10^7copies/μl	15.58	0.56	1.69
		10^6copies/μl	19.23	1.70	2.97
		10^5copies/μl	22.61	1.56	2.57
		10^4copies/μl	26.32	0.66	1.36
		10^3copies/μl	29.93	0.30	0.84
		10^2copies/μl	33.56	1.23	1.39
		10^1copies/μl	36.72	1.07	0.52
	SFTSV	10^8copies/μl	11.06	1.64	2.17
		10^7copies/μl	14.95	0.77	1.11
		10^6copies/μl	18.7	1.63	1.37
		10^5copies/μl	22.16	0.71	0.69
		10^4copies/μl	26.09	0.03	0.16
		10^3copies/μl	29.62	0.23	0.58
		10^2copies/μl	32.89	0.54	0.41
		10^1copies/μl	35.96	1.50	0.18
	HLV	10^8copies/μl	9.86	0.20	0.71
		10^7copies/μl	14.08	2.82	3.00
		10^6copies/μl	17.42	0.21	3.87
		10^5copies/μl	21.22	0.34	2.84
		10^4copies/μl	25.08	0.16	1.80
		10^3copies/μl	28.86	0.52	1.21
		10^2copies/μl	32.5	0.16	1.37
		10^1copies/μl	36.14	1.18	0.34
Group D	OHFV	10^8copies/μl	10.98	1.95	0.44
		10^7copies/μl	14.61	0.91	2.66
		10^6copies/μl	18.21	0.43	3.27
		10^5copies/μl	21.80	0.31	2.10
		10^4copies/μl	25.31	0.40	2.41
		10^3copies/μl	28.96	0.32	1.11
		10^2copies/μl	32.28	0.43	1.63
		10^1copies/μl	35.86	1.05	0.51
	KFDV	10^8copies/μl	11.54	2.37	0.30
		10^7copies/μl	15.13	0.33	0.87
		10^6copies/μl	18.72	0.93	1.48
		10^5copies/μl	22.1	0.09	1.04
		10^4copies/μl	25.42	0.27	2.11
		10^3copies/μl	28.6	0.19	2.41
		10^2copies/μl	31.21	0.46	1.73
		10^1copies/μl	35.03	3.01	3.02
	DENV	10^8copies/μl	11.50	3.66	4.65
		10^7copies/μl	15.28	0.26	1.91
		10^6copies/μl	18.70	0.64	1.81
		10^5copies/μl	22.28	0.10	1.07
		10^4copies/μl	25.99	0.25	1.13
		10^3copies/μl	29.46	0.21	1.14
		10^2copies/μl	33.00	0.33	0.88
		10^1copies/μl	36.15	1.10	1.05

	YFV	10^8copies/μl	10.72	2.84	3.11
		10^7copies/μl	14.35	2.49	2.36
		10^6copies/μl	17.70	1.40	1.53
		10^5copies/μl	20.61	0.97	2.60
		10^4copies/μl	23.96	0.50	2.88
		10^3copies/μl	27.67	0.71	2.45
		10^2copies/μl	31.19	0.18	1.80
		10^1copies/μl	34.65	1.34	1.66
Group E	MARV	10^8copies/μl	10.28	2.81	3.48
		10^7copies/μl	14.27	1.54	1.07
		10^6copies/μl	17.76	1.056	1.83
		10^5copies/μl	21.29	1.08	0.99
		10^4copies/μl	24.88	0.39	1.41
		10^3copies/μl	28.45	0.24	0.92
		10^2copies/μl	31.70	0.50	1.03
		10^1copies/μl	34.97	0.35	1.33
	ZEBOV	10^8copies/μl	10.75	3.48	3.17
		10^7copies/μl	15.31	0.28	4.58
		10^6copies/μl	18.80	0.26	3.26
		10^5copies/μl	22.47	0.45	2.57
		10^4copies/μl	25.97	0.23	2.17
		10^3copies/μl	29.36	0.33	1.49
		10^2copies/μl	32.91	0.32	1.33
		10^1copies/μl	36.48	2.34	2.26
	SEBOV	10^8copies/μl	10.02	0.51	0.63
		10^7copies/μl	13.77	0.63	1.40
		10^6copies/μl	17.34	0.30	2.09
		10^5copies/μl	20.88	0.72	1.17
		10^4copies/μl	24.39	0.49	2.39
		10^3copies/μl	27.85	0.26	3.23
		10^2copies/μl	31.34	0.37	2.34
		10^1copies/μl	34.77	0.62	1.71
	CEBOV	10^8copies/μl	10.31	3.22	3.03
		10^7copies/μl	14.27	0.97	0.48
		10^6copies/μl	17.79	0.31	0.847
		10^5copies/μl	21.18	0.20	1.15
		10^4copies/μl	24.95	0.34	0.34
		10^3copies/μl	28.24	0.45	0.91
		10^2copies/μl	31.46	1.06	1.42
		10^1copies/μl	34.97	0.80	0.84
Group F	JUNV	10^8copies/μl	11.59	1.91	4.41
		10^7copies/μl	15.03	0.29	2.91
		10^6copies/μl	18.78	1.16	2.29
		10^5copies/μl	22.15	0.63	0.73
		10^4copies/μl	25.48	0.10	0.08
		10^3copies/μl	29.12	0.29	0.46
		10^2copies/μl	32.87	0.36	0.46
		10^1copies/μl	36.61	1.31	2.01
	MACV	10^8copies/μl	10.28	0.90	3.80
		10^7copies/μl	14.04	0.33	0.50
		10^6copies/μl	17.47	0.72	0.49
		10^5copies/μl	20.91	1.22	1.31
		10^4copies/μl	24.37	1.21	2.21
		10^3copies/μl	28.01	0.72	0.75
		10^2copies/μl	31.66	0.63	0.53
		10^1copies/μl	35.33	1.73	1.69
	GTOV	10^8copies/μl	10.64	0.85	1.80
		10^7copies/μl	14.65	0.75	0.76
		10^6copies/μl	17.84	1.70	2.47
		10^5copies/μl	21.57	1.01	2.02
		10^4copies/μl	25.26	0.38	2.17
		10^3copies/μl	28.69	0.55	1.93
		10^2copies/μl	32.37	0.36	1.27
		10^1copies/μl	35.55	2.10	1.88

	SABV	10^8copies/μl	10.64	1.85	3.03
		10^7copies/μl	14.42	0.60	2.52
		10^6copies/μl	17.98	0.64	0.07
		10^5copies/μl	21.44	1.15	0.76
		10^4copies/μl	25.05	0.08	0.70
		10^3copies/μl	28.57	0.43	1.13
		10^2copies/μl	32.11	0.31	0.74
		10^1copies/μl	34.99	1.76	0.87
Group G	CHAV	10^8copies/μl	11.13	2.20	4.52
		10^7copies/μl	14.64	0.45	2.89
		10^6copies/μl	18.28	0.35	1.89
		10^5copies/μl	21.71	0.34	0.96
		10^4copies/μl	25.39	0.06	0.86
		10^3copies/μl	28.95	0.19	1.13
		10^2copies/μl	32.31	0.60	1.26
		10^1copies/μl	35.87	1.10	1.44
	LASV	10^8copies/μl	10.93	3.97	4.53
		10^7copies/μl	14.62	1.22	1.08
		10^6copies/μl	18.29	0.13	0.32
		10^5copies/μl	21.48	0.51	0.99
		10^4copies/μl	24.77	0.62	1.18
		10^3copies/μl	28.22	0.24	1.69
		10^2copies/μl	31.59	0.58	2.03
		10^1copies/μl	34.88	0.64	2.59
	LUJV	10^8copies/μl	10.63	3.90	2.87
		10^7copies/μl	14.89	4.38	4.10
		10^6copies/μl	17.84	1.69	2.15
		10^5copies/μl	21.52	1.22	1.71
		10^4copies/μl	25.44	2.09	2.08
		10^3copies/μl	28.71	0.76	0.85
		10^2copies/μl	32.13	2.31	1.26
		10^1copies/μl	35.44	1.46	0.44
	BASV	10^8copies/μl	11.83	0.63	4.98
		10^7copies/μl	15.22	0.42	3.72
		10^6copies/μl	18.87	0.55	2.49
		10^5copies/μl	22.61	1.02	2.41
		10^4copies/μl	26.08	0.23	1.97
		10^3copies/μl	29.73	1.47	1.98
		10^2copies/μl	33.21	0.69	0.75
		10^1copies/μl	36.62	1.70	1.90

CV, coefficient of variation.

Intra-assays were determined from three replicates within each dilution.

Inter-assays were determined from three independent assays performed on different days.
